# Finite Element Analysis and Experimental Investigation on the Machinability of PMMA/CNT Composites via Nanosectioning

**DOI:** 10.3390/polym17182441

**Published:** 2025-09-09

**Authors:** Guoyu Fu, Jia Ge, Hao Li, Fengzhen Sun, Weizhou Wu

**Affiliations:** 1 Key Laboratory of Mechanism Theory and Equipment Design of Ministry of Education, Tianjin University, Tianjin 300072, China; guoyu07@tju.edu.cn (G.F.); haolitju@tju.edu.cn (H.L.); wwxzz@tju.edu.cn (W.W.); 2 School of Mechanical and Aerospace Engineering, Queen’s University Belfast, Belfast BT9 5AH, UK; jge02@qub.ac.uk; 3 School of Mechanical Engineering, Tongji University, 4800 Cao’an Road, Shanghai 201804, China

**Keywords:** nanosectioning, carbon nanotubes (CNTs), polymer nanocomposites, Mulliken–Boyce model

## Abstract

In this study, an innovative modeling approach has been proposed to demonstrate the removal mechanisms of Polymethyl Methacrylate (PMMA) reinforced with randomly distributed carbon nanotubes (CNTs) during nanosectioning. The viscoplastic behavior of the matrix polymer was described using the Mulliken–Boyce model and the distribution of the CNTs in the matrix was modeled using the random sequential adsorption (RSA) method. The effects of cutting thickness and CNT loading on the machinability of the nanocomposites are explored. Subsequent experiments were conducted to validate the modeling. It reveals that the addition of CNT increases the resistance to cutting, compared to the malleable matrix. Although the primary strain distribution for both plain PMMA and PMMA/CNT composites aligns closely, discernible disparities between the two materials emerge. A force augmentation is anticipated whenever a nanotube interacts with the cutting tool, which causes surface protrusions and sub-surface damage. The addition of CNT with a loading lower than 1.0 wt% does not change the mechanisms of chip formation, but the addition of 1.0 wt% CNTs increases cutting force by approximately 32%. This work provides a feasible approach and framework to numerically model the nanosectioning of CNT-reinforced thermoplastics.

## 1. Introduction

Carbon nanotubes (CNTs), encompassing single- and multi-wall variants, are cylindrical nanostructures derived from SP2 hybridized carbon atoms [1,2]. These nanostructures, with diameters ranging from 1 to 100 nm and lengths from 0.1 to 100 µm, are formed by rolling up single layers of carbon atoms [3]. Due to their diminutive size, high aspect ratio, and excellent mechanical and electrical properties, CNTs have become a leading reinforcement for polymers. Polymer/CNT nanocomposites have gained significant traction due to advantages such as ease of fabrication, enhanced processing capabilities, durability, and cost benefits over metals and ceramics [4,5,6], apart from their superior mechanical, thermal, and electrical properties [7,8,9,10,11,12,13,14].

Numerous studies have been conducted to investigate the machinability of polymer/CNT nanocomposites. Kumar et al. [15] showed that the introduction of CNTs can significantly improve the dimensional accuracy and quality of micro-machined surfaces. Mahmoudi et al. [16] showed that the machinability of polymer/CNT nanocomposites was better than that of plain polymers due to improved thermal conductivity. Samuel et al. [17] found the ductile-to-brittle transition that occurred with an increase in CNT loading. Gong et al. [18] quantified the relation between surface roughness and cutting parameters of polymer/CNT nanocomposites; for instance, the surface roughness exhibits a linear dependency upon the feed rate and a quadratic dependency upon the cutting speed. These studies predominantly focus on macro- and micro-machining, and thus there is a noticeable gap in the research concerning the nanomachining of polymer/CNT nanocomposites. While macro- and micro-machining share certain kinematic similarities, nanomachining presents distinct challenges due to the size effect [19,20,21]. In particular, when the feed per tooth (FPT) is too low, a chip cannot form during each cutting [22,23]. As the FPT value approaches the radius of the tool, the material undergoes intense ploughing/friction during each cutting [24,25,26]. In such cases, the tool primarily displaces the material, with elastic recovery dominating the deformation [27,28]. When the cutting thickness falls below a threshold, the size effect introduces adverse impacts on the cutting process, such as elevated cutting forces, reduced tool lifespan, and irregular surface topography [29,30].

Because of the large plastic deformation, thermoplastic coupling, and reinforcement-knife contact mechanisms occurring in the vicinity of the knife edge, it is very challenging to probe the machining process via experiment. Therefore, finite element (FE) modeling is extensively adopted to probe the material responses during the machining process. However, there are few reports on modeling polymer/CNT machining due to the challenges in modeling the viscoplastic behavior of polymers and integrating the CNTs into the matrix. This study aims to address a notable gap concerning the simulation and experimental machining of polymer/CNT nanocomposites. We conduct FE modeling of the machining of polymer/CNT nanocomposites using the Mulliken–Boyce model, in combination with a random distribution algorithm to integrate CNTs into the matrix material. This approach offers a useful perspective on simulating the machinability of CNT nanocomposites, different from the conventionally adopted molecular dynamics methods [31]. The FE cutting model has proven instrumental in exploring the machining mechanisms of polymer/CNT nanocomposites, particularly emphasizing their mechanical attributes and response. The modeling results were then validated with experimentally measured cutting force and chip morphology.

## 2. Preparation of Nanocomposites

### 2.1. Materials and Preparation

Commercial multi-walled CNTs (MWCNTs) were sourced from Cheap Tubes Inc., Grafton, VT, USA. These MWCNTs, produced via chemical vapor deposition, possess an outer diameter of 30–35 nm, an inner diameter of 5–10 nm, a length ranging from 1 to 6 μm, and a purity exceeding 95%. The polymethyl methacrylate (PMMA) used was the commercial-grade H15 002 polymer obtained from Plastiglas, Ocoyoacac, Estado de México, Mexico. A standard reagent, chloroform, boasting a purity of 98.8%, was employed in the fabrication.

Both plain PMMA and PMMA/MWCNT films were synthesized using the solution casting method. For the preparation of nanocomposites with specified MWCNT contents, PMMA was dissolved in chloroform with the aid of a mechanical stirrer. Concurrently, MWCNTs were dispersed in chloroform using ultrasonication for an hour. Following the ultrasonic dispersion of MWCNTs, the two solutions were amalgamated under a laboratory hood and continuously stirred at 70 °C, allowing the solvent to evaporate over a span of 20 min.

To enhance MWCNT dispersion, two additional ultrasonic stirring cycles were executed prior to casting: an initial 15 min cycle followed by a 10 min cycle. The resulting viscous mixture was then cast into a glass mold, where the solvent was allowed to evaporate gradually over 24 h, culminating in the formation of a solid film with a thickness approaching 300 μm. The curing process was conducted in a convection oven and comprised three sequential stages: 60 °C, 70 °C, and 80 °C, with each stage lasting 24 h. This was to ensure the complete removal of any residual solvent. Using this methodology, composites with MWCNT concentrations of 0.1 wt%, 0.2 wt%, 0.5 wt%, and 1.0 wt% were fabricated. Plain PMMA films were synthesized following a similar protocol.

Achieving a uniform distribution of CNTs in PMMA has always been a critical challenge in experimental studies. Several methods have been proposed and shown to be effective in overcoming this issue. First, ultrasound dispersion techniques are widely used to disperse CNTs, where high-frequency vibrations help reduce CNT agglomeration in solvents. However, excessive ultrasound treatment time and energy may lead to fractures or chemical structural changes of CNTs, so careful control of the process is necessary [32]. Second, surface functionalization of CNTs, such as oxidation or grafting functional groups (e.g., carboxyl or amino groups), significantly improves the compatibility between CNTs and the PMMA matrix, thus promoting their uniform distribution [33]. Additionally, solvent system optimization and solution immersion methods, by choosing appropriate solvents and adjusting concentration and temperature, help optimize CNT dispersion. Electrostatic or mechanical field-assisted dispersion has also been applied, where electric fields aid in the orientation of CNTs, effectively reducing aggregation [34]. Combining and optimizing these methods can address the challenge of uniform CNT distribution in PMMA, thereby improving the mechanical and processing performance of the composite material.

### 2.2. Carbon Nanotube Distribution

**Figure 1:**
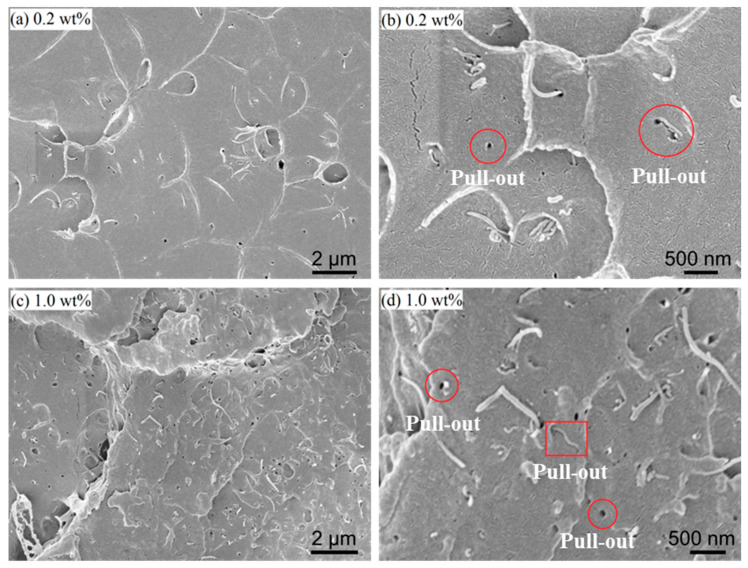
SEM micrographs of fracture surfaces of PMMA/MWCNT nanocomposites. (**a**,**b**) 0.2 wt%: low-magnification view and higher-magnification view highlighting nanotube pull-out. (**c**,**d**) 1.0 wt%: low-magnification view and higher-magnification view showing nanotube pull-out accompanied by interfacial debonding.

Prior to nanosectioning, we characterized the dispersion state and interfacial features of MWCNTs in PMMA by scanning electron microscopy (SEM). Specimens were fractured in bending and the fracture surfaces were sputter-coated with ~40 nm Au to ensure conductivity. Representative micrographs for 0.2 wt% and 1.0 wt% MWCNTs are shown in Figure 1. SEM fracture-surface morphologies are shown in Figure 1a–d: nanotube pull-out at 0.2 wt% (Figure 1a,b) and pull-out with interfacial debonding at 1.0 wt% (Figure 1c,d). At both loadings, nanotubes are embedded within the matrix with tortuous, non-collinear trajectories, consistent with a predominantly random in-plane orientation rather than long-range alignment. Two dominant interfacial failure signatures are evident: (i) nanotube pull-out leaving cylindrical voids, indicative of interfacial sliding; and (ii) groove-like debonding traces when nanotubes lie nearly parallel to the fracture plane, reflecting local loss of adhesion. Occasional defects—interfacial gaps at the tube–matrix boundary, isolated porosity, and fine matrix cracks—are also observed. These microstructural features substantiate the modeling assumptions used in our FE framework: a random spatial distribution of short inclusions (as generated by RSA) and the possibility of interfacial separation/pull-out under load transfer. They also provide a mechanistic basis for the measured force fluctuations during nanosectioning, where local tube–tool encounters and interfacial debonding can transiently elevate the cutting resistance.

## 3. Experimental Sectioning

**Figure 2:**
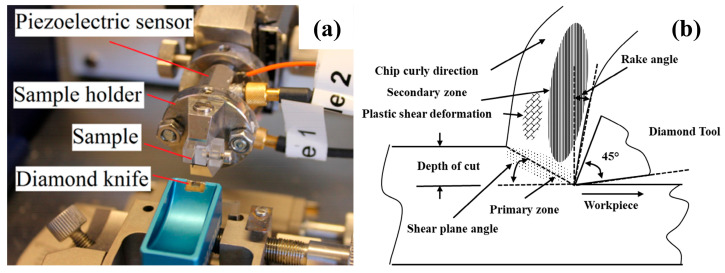
Ultramicrotome setup and orthogonal cutting schematic. (**a**) Instrument with piezoelectric sensor, holder, sample, and diamond knife. (**b**) 45° rake, depth of cut, shear-plane angle, primary/secondary zones, and chip curl.

Figure 2 illustrates the ultramicrotome used for conducting the nanosectioning, which is equipped with two piezoelectric load cells (PCB 209A12). Each specimen undergoes continuous slicing at a consistent rate of 1.0 mm/s, with depths set at 60 nm, 80 nm, 100 nm, 120 nm, 150 nm, and 200 nm. The slicing chips are observed using a CCD eyepiece camera. Additionally, the dimensions of the chips, in terms of length and width, are measured before and after slicing.

## 4. Modeling of Sectioning of PMMA/CNT

### 4.1. Mulliken–Boyce Model

**Figure 3:**
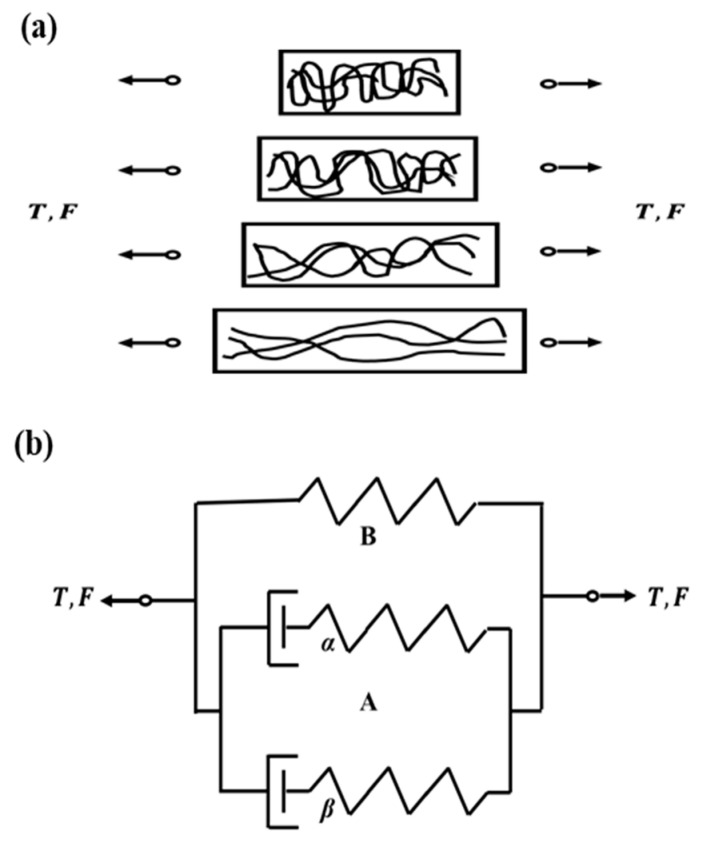
(**a**) Rotation of the molecules of polymer material during deformation; (**b**) one-dimensional rheological interpretation of the proposed constitutive model [37].

To investigate the sectioning behavior of polymers, this study adopts the Mulliken–Boyce model [35,36] to describe the mechanical response of PMMA under varying strain rates, which was developed based on the Arruda–Boyce model [30]. The Mulliken–Boyce model is a physics-driven constitutive model rooted in the theory of extensive plasticity. It models the elasticity, yield, and post-yield behavior of polymers across a spectrum of strain rates [37]. Comprising two activated molecular processes that run in parallel, as well as the nonlinear entropy hardening process [37]. Figure 3 provides a one-dimensional rheological representation of the Mulliken–Boyce model.

### 4.2. Kinematics

In the Mulliken–Boyce model [35], the deformation gradient, *F*, which maps a material point from its reference position *X* to the current location x, is defined as

(1) $$F\equiv \frac{x}{X}$$

During loading, all parallel phases undergo the same deformation:

(2) $$F\equiv F_{A_{\alpha }}=F_{A_{\beta }}=F_{B}$$

The deformation gradient in elements α and β is multiplicatively decomposed into elastic and plastic components via Kroner–Lee decomposition:

(3) $$F_{A_{\alpha }}=F_{A_{\alpha }}^{e}F_{A_{\alpha }}^{p}$$

(4) $$F_{A_{\beta }}=F_{A_{\beta }}^{e}F_{A_{\beta }}^{p}$$

Assuming that the plastic deformation is a volume-preserving process, then

(5) $$\det F_{A_{\alpha }}^{p}=\det F_{A_{\beta }}^{p}=1$$

All volume-changing deformation is elastic:

(6) $$\det F_{A_{\alpha }}^{e}=\det F_{A_{\beta }}^{e}=J$$

Therefore, the deformation of the relaxed configuration can be obtained as

(7) $$F_{A_{\alpha }}^{p}=R_{A_{\alpha }}^{p}U_{A_{\alpha }}^{p}=V_{A_{\alpha }}^{p}U_{A_{\alpha }}^{p}$$

(8) $$F_{A_{\beta }}^{p}=R_{A_{\beta }}^{p}U_{A_{\beta }}^{p}=V_{A_{\beta }}^{p}U_{A_{\beta }}^{p}$$

The velocity gradients including elastic and plastic components are defined as

(9) $$L_{A_{\alpha }}=L_{A_{\alpha }}^{p}+F_{A_{\alpha }}^{e}L_{A_{\alpha }}^{p}F_{A_{\alpha }}^{e-1}$$

and

(10) $$L_{A_{\beta }}=L_{A_{\beta }}^{p}+F_{A_{\beta }}^{e}L_{A_{\beta }}^{p}F_{A_{\beta }}^{e-1}$$

where

(11) $$L_{A_{\alpha }}^{p}={\dot{F}}_{A_{\alpha }}^{p}F_{A_{\alpha }}^{p-1}=D_{A_{\alpha }}^{p}+W_{A_{\alpha }}^{p}$$

(12) $$L_{A_{\beta }}^{p}={\dot{F}}_{A_{\beta }}^{p}F_{A_{\beta }}^{p-1}=D_{A_{\beta }}^{p}+W_{A_{\beta }}^{p}$$

The plastic flow is irrotational in both the *α* and *β* components:

(13) $$W_{A_{\alpha }}^{p}+W_{A_{\beta }}^{p}=0$$

and therefore

(14) $${\dot{F}}_{A_{\alpha }}^{p}=D_{A_{\alpha }}^{p}F_{A_{\alpha }}^{p}$$

(15) $${\dot{F}}_{A_{\beta }}^{p}=D_{A_{\beta }}^{p}F_{A_{\beta }}^{p}$$

Then integrated to derive $F_{A_{\alpha }}^{p}$ and $F_{A_{\beta }}^{p}$, and the elastic deformation gradients of the polymer can be obtained via

(16) $$F_{A_{\alpha }}^{e}=F_{A_{\alpha }}F_{A_{\alpha }}^{p-1}$$

(17) $$F_{A_{\beta }}^{e}=F_{A_{\beta }}F_{A_{\beta }}^{p-1}$$

Finally, the plastic flow rules are governed by

(18) $$D_{A_{\alpha }}^{p}={\dot{\gamma }}_{A_{\alpha }}^{p}N_{A_{\alpha }}^{p}$$

(19) $$D_{A_{\beta }}^{p}={\dot{\gamma }}_{A_{\beta }}^{p}N_{A_{\beta }}^{p}$$

where

(20) $$N_{A_{\alpha }}^{p}=\frac{T_{A_{\alpha }}^{\prime }}{\left|T_{A_{\alpha }}^{\prime }\right|}$$

(21) $$N_{A_{\beta }}^{p}=\frac{T_{A_{\beta }}^{\prime }}{\left|T_{A_{\beta }}^{\prime }\right|}$$

### 4.3. Material Description and Constitutive Relations

The intermolecular contribution to the material stress state is related to the deformation according to the constitutive laws for linear elastic springs:

(22) $$T_{A_{\alpha }}=\frac{1}{J_{\alpha }}{ℒ}_{\alpha }^{e}\left[InU_{A_{\alpha }}^{e}\right]$$

(23) $$T_{A_{\beta }}=\frac{1}{J_{\beta }}{ℒ}_{\beta }^{e}\left[InU_{A_{\beta }}^{e}\right]$$

where $T_{A_{i}}(i=\alpha ,\beta )$ is the Cauchy stress, ${ℒ}_{i}^{e}$ is the fourth-order modulus tensor, and ln$U_{A_{i}}^{e}$ is the Hencky strain. Assuming the polymer is initially isotropic and that the elastic behavior of the polymer can be decomposed into *α* and *β* components, the modulus tensor is derived from the shear modulus µ and bulk modulus κ:

(24) $${ℒ}_{\alpha }^{e}=2\mu_{\alpha }ℐ+(\kappa_{\alpha }-\frac{2}{3}\mu_{\alpha })Ι⊗Ι$$

(25) $${ℒ}_{\beta }^{e}=2\mu_{\beta }ℐ+(\kappa_{\beta }-\frac{2}{3}\mu_{\beta })Ι⊗Ι$$

where ℐ and Ι are the fourth- and second-order identity tensors, respectively. $\mu_{i}$ and $\kappa_{i}$ (*i* = *α*, *β*), are assumed to be functions of both temperature and strain rate.

The stress in the nonlinear hardening component, representing the network ‘back stress’ due to molecular alignment, is defined as

(26) $$T_{B}=\frac{C_{R}}{3}\frac{\sqrt{N}}{\lambda_{chain}^{p}}{ℒ}^{-1}\left(\frac{\lambda_{chain}^{p}}{\sqrt{N}}\right)B_{B}^{\prime }$$

where $\lambda_{chain}^{p}$ is the stretch on a chain in the eight-chain network; ℒ is the Langevin function defined by $ℒ\left(\beta \right)\equiv \coth\beta -\frac{1}{\beta }$; $B_{B}^{\prime }$ is the deviatoric part of the left Cauchy–Green tensor, $F_{B}F_{B}^{T}$; $\sqrt{N}$ is the limiting chain extensibility; and $C_{R}\equiv nk\theta$ is the rubbery modulus.

The total stress in the polymer is the tensorial sum of the α and β intermolecular stresses and the back stress:

(27) $$T=T_{A_{\alpha }}+T_{A_{\beta }}+T_{B}$$

The driving stresses are used to specify the direction tensors $N_{A_{\alpha }}^{p}$ and $N_{A_{\beta }}^{p}$ in the flow rules:

(28) $$N_{\alpha }=\frac{1}{\sqrt{2}\tau_{\alpha }}T_{A_{\alpha }}^{\prime }$$

(29) $$N_{\beta }=\frac{1}{\sqrt{2}\tau_{\beta }}T_{A_{\beta }}^{\prime }$$

The effective equivalent shear stresses $\tau_{\alpha }$ and $\tau_{\beta }$ are defined as

(30) $$\tau_{\alpha }=\sqrt{\frac{1}{2}T_{A_{\alpha }}^{\prime }T_{A_{\alpha }}^{\prime }}$$

(31) $$\tau_{\beta }=\sqrt{\frac{1}{2}T_{A_{\beta }}^{\prime }T_{A_{\beta }}^{\prime }}$$

They modified the flow rule in the Mulliken–Boyce model [38] by including new internal variables to characterize viscoplastic behavior:

(32) $${\dot{\gamma }}_{\alpha }^{p}={\dot{\gamma }}_{0,\alpha }^{p}\exp\left[-\frac{\Delta G_{\alpha }}{k\theta }\left(1-\frac{\tau_{\alpha }}{s_{\alpha }+\alpha_{\alpha +\beta }p}\right)\right]$$

(33) $${\dot{\gamma }}_{\beta }^{p}={\dot{\gamma }}_{0,\beta }^{p}\exp\left[-\frac{\Delta G_{\beta }}{k\theta }\left(1-\frac{\tau_{\alpha }}{s_{\beta }+\alpha_{\alpha +\beta }p}\right)\right]$$

where ${\dot{\gamma }}_{0,i}^{p}$ (i=α,β) is the factor proportional to the attempt frequency, $\Delta G_{i}$ is the activation energy, p is the pressure, and $\alpha_{p,i}p$ is the pressure coefficient. The internal variable $s_{i}$ is the shear strength, which is related to the shear modulus and evolves to a preferred state with plastic straining:

(34) $$s_{0,\alpha }\equiv \frac{0.077\mu_{\alpha }}{1-\nu_{\alpha }}$$

(35) $${\dot{s}}_{\alpha }=h_{\alpha }\left(1-\frac{s_{\alpha }}{s_{ss,a}}\right){\dot{\gamma }}_{\alpha }^{p}$$

(36) $$s_{0,\beta }\equiv \frac{0.077\mu_{\beta }}{1-\nu_{\beta }}$$

(37) $${\dot{s}}_{\beta }=h_{\beta }\left(1-\frac{s_{\beta }}{s_{ss,\beta }}\right){\dot{\gamma }}_{\beta }^{p}$$

where $h_{i}$ (i=α,β) represents the softening slope, and $s_{ss,i}$ is termed the ‘preferred state’. This internal variable is designed to allow the yield stress’s temperature dependence to mirror that of the elastic shear moduli, effectively capturing the strain-softening phenomenon. In the most comprehensive rendition of this constitutive model, the strain-softening effect can be interpreted as the cumulative softening of both the α and β components. During FE Analysis, CNTs were assumed to undergo brittle fracture [39].

### 4.4. Parameters for PMMA

A one-dimensional Mulliken–Boyce model used genetic algorithms to deduce the constitutive parameters that govern the stress-strain behavior of PMMA [40]. In this work, these parameters were adopted by the 3D model and are detailed in Table 1.

### 4.5. Sectioning Model

A microstructural cutting model was devised, which integrates microstructural representations with the corresponding constitutive and failure models. The matrix material predominantly exhibits ductile failure. The chip formation process in PMMA/CNT nanocomposites during cutting can be linked to a ductile failure mechanism. The failure of temperature-sensitive PMMA is described using the Gearing–Anand model [42]. For the MWCNTs, a strain failure criterion is employed.

**Figure 4:**
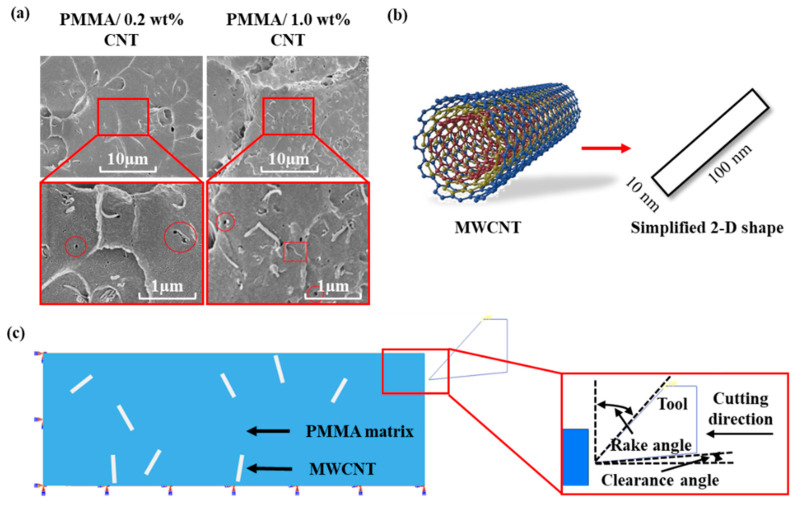
(**a**) SEM micrographs of PMMA/CNTs fracture surfaces [49], (**b**) simplification of MWCNT into 2D rectangular shape [50], and (**c**) schematics showing the FE set-up for orthogonal cutting of PMMA/CNTs.

Figure 4a presents SEM images of the fractured surfaces of PMMA/MWCNT composites. Figure 4b illustrates a streamlined modeling approach for the PMMA/MWCNT nanocomposites, while Figure 4b,c depict the FE configuration for orthogonal cutting of the nanocomposites. The FE model is distilled into a 2-phase representation. CNTs disperse within the matrix material randomly, which are simplified as rectangles with dimensions of 100 nm × 10 nm. A comparison model devoid of CNTs serves as a control. The cutting tool, assumed to be an analytical rigid body, traverses a stationary workpiece at a defined velocity. In the cutting model, the polymer and CNT geometries are generated independently, and then are assembled with their interfaces integrated seamlessly. The matrix and CNTs are meshed independently, ensuring no shared nodes. The density, Young’s modulus, shear modulus, Poisson’s ratio, critical stress intensity factor, and equivalent critical strain energy release rate of CNT are specified as 2.267 g/cm^3^ [43], 1 TPa [44], 280 GPa [45], −0.38 [46], 4.0 MPa$\sqrt{m}$ [47], and 15.9 Jm^−2^ [48], respectively.

Table 2 documents the parameters of the cutting model and conditions employed in this study. The cutting speed is set at 1.0 mm/s, with six distinct cutting thicknesses ranging from 60 nm to 200 nm. The tool rake and clearance angles are 45.0° and 5.0°, respectively. The model dimensions are 1.6 µm by 0.5 µm. Drawing from the density metrics of PMMA and CNTs, the reinforcement fractions in the FE simulation are 0.05%, 0.1%, 0.25%, and 0.5%.

### 4.6. Distribution of CNTs

**Figure 5:**
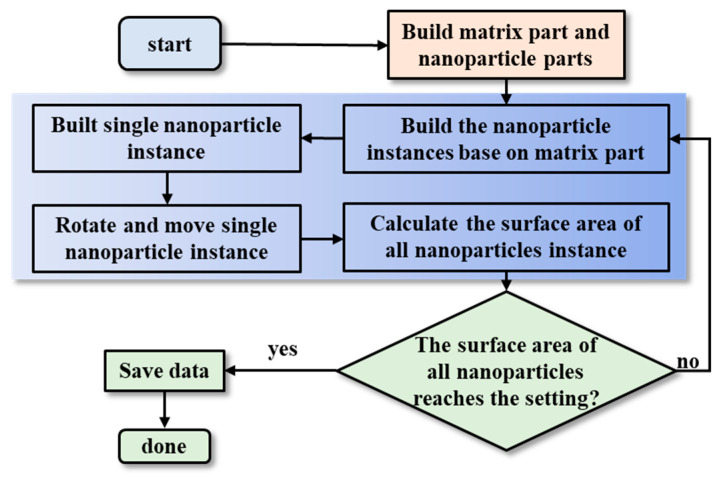
Flow chart of the RVE generation algorithm for polymer/CNTs nanocomposites.

This study employs the random sequential adsorption (RSA) method as its primary algorithm for the random distribution of MWCNTs [51,52]. Figure 5 displays the flow chart of the RSA algorithm tailored for polymer/CNT nanocomposites. The program ceases once the cumulative area fraction of the nanotubes achieves a threshold.

In its foundational design, the RSA algorithm iteratively introduces MWCNTs into the matrix by stochastically determining the locations for inclusions. Initially, distinct entities for the matrix and nanotubes are constructed. Subsequent to this, instances of nanotubes are instantiated, with their spatial coordinates and orientations being randomized. The algorithm then assesses for potential overlaps between nanotubes. In the event of an overlap, the conflicting nanotube undergoes random repositioning and reorientation until it is free from intersections. Once a nanotube is successfully placed without conflicts, the algorithm proceeds to introduce the next one, and the assessment cycle recommences.

**Figure 6:**
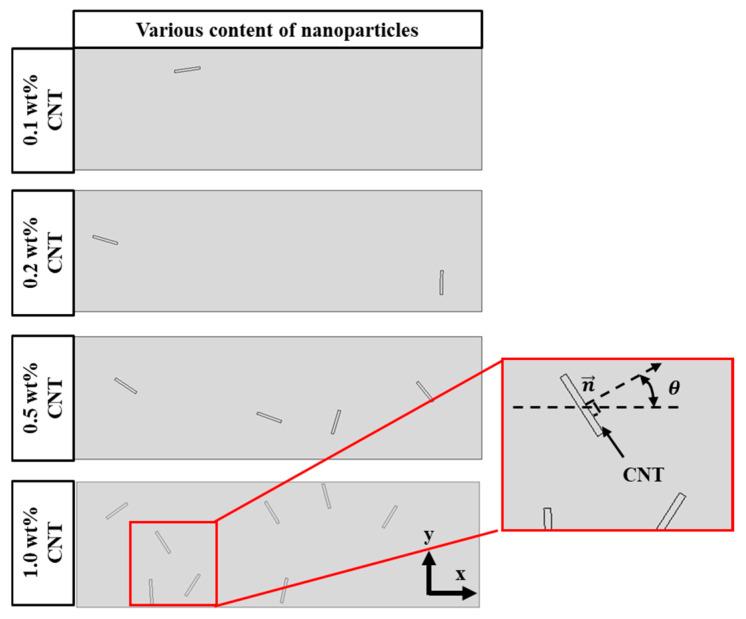
Various contents of CNT in nanocomposites in the 2D micro-FE cutting model.

Figure 6 shows various contents of CNTs in nanocomposites for 2D FE cutting models. The probability of the random nanotube distribution is denoted as $P_{0}$. As shown in Figure 5, θ is the disorientation angle between the normal vector ($\vec{n}$) of the nanotube and the x axis, which can describe the orientation of the CNT in the matrix. It is obvious that when θ is equal to 0 and π/2, $P_{0}$ is equal to 1 and 0, respectively. Thus, $P_{0}$ can be assumed to be a function of θ as follows [53,54]:

(38) $$P_{0}=(1-\frac{2}{\pi }\theta )$$

## 5. Results and Discussion

### 5.1. Cutting Force

**Figure 7:**
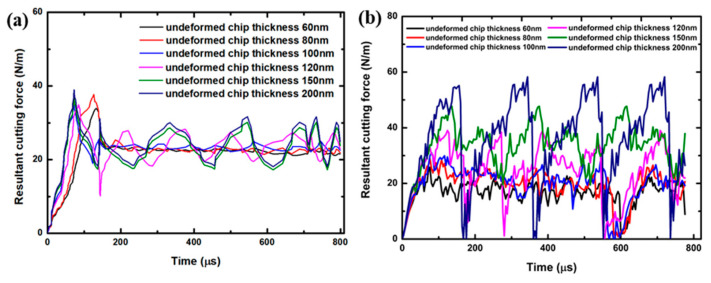
(**a**) Experimental and (**b**) simulated cutting force of plain PMMA at various cutting thicknesses under 1 mm/s cutting.

Figure 7 shows the simulated cutting force from the various composites and plain PMMA, and the measurement of resultant cutting force is used to evaluate the machinability. Using the computed thrust force per width, $F_{t}$, and the cutting force per width, $F_{c}$, the specific cutting energy, F, is calculated as [55]:

(39) $$F=\sqrt{F_{t}^{2}+F_{c}^{2}}$$

Figure 7a illustrates the experimental cutting force for plain PMMA at various cutting thicknesses under a cutting speed of 1 mm/s. The results demonstrate that the cutting forces increase with the cutting thickness, with lower thicknesses such as 60 nm and 80 nm producing relatively stable force trends, while higher thicknesses such as 150 nm and 200 nm exhibit larger force magnitudes and variability. The force profile initially shows a surge, referred to as the entry shock, which corresponds to the tool engaging with the material [56]. Following this phase, the forces transition to a steady-state region with occasional fluctuations, reflecting dynamic tool-material interactions during sectioning.

Figure 7b depicts the simulated cutting force for plain PMMA across various cutting thicknesses at a cutting speed of 1 mm/s. The cutting settings for both the FE simulation and the experiment are the same. The cutting forces exhibit an initial surge, termed the entry shock, which then transitions to a steady-state phase. Notably, even during this steady-state phase, the forces display fluctuations. As the tool progresses, there is an escalation in the cutting force, leading to heightened plastic deformation. Consequently, a decline in the cutting force is observed when the tool traverses the previously softened segment of the material. Upon reaching this juncture, the cycle of force fluctuations resets, and the pattern recurs with the tool’s forward movement. It is noteworthy that as the cutting depth increases, the force fluctuations become increasingly pronounced.

**Figure 8:**
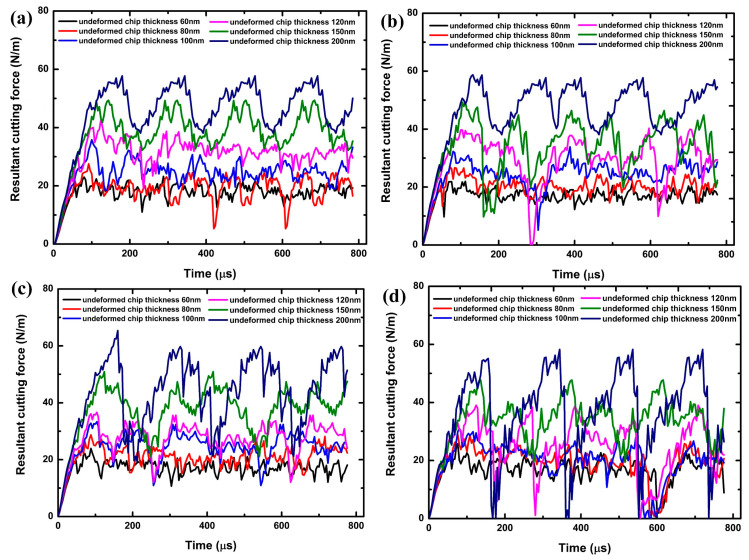
Simulated resultant cutting force of PMMA/CNT nanocomposites at various cutting thicknesses under 1 mm/s cutting speed: (**a**) PMMA/0.1 wt% CNTs; (**b**) PMMA/0.2 wt% CNTs; (**c**) PMMA/0.5 wt% CNTs; (**d**) PMMA/1.0 wt% CNTs.

Figure 8 presents the simulated resultant cutting force for various PMMA/CNT nanocomposites across different cutting thicknesses at a cutting speed of 1 mm/s. The steady-state cutting forces observed in the composite exhibit fluctuations akin to those in pure PMMA. However, these fluctuations in the composites are notably more pronounced and irregular compared to those in standard PMMA. This can be ascribed to the microstructural heterogeneity introduced by the CNTs. The inherently robust CNTs pose greater resistance to cutting or displacement than the comparatively malleable PMMA matrix. As a result, any interaction of a CNT with the cutting tool is likely to induce a spike in the cutting force. The magnitude and direction of this effect are also contingent on the orientation and length of the CNT [57]. Thus, the observed fluctuations in the steady-state force emerge from the combined influence of CNTs within the PMMA matrix. The FE simulation results suggest that the spatial distribution of CNTs and their concentration significantly impact both the cutting force and chip formation. These force fluctuations are intrinsically tied to the chip formation process. It is worth noting that the forces associated with pure polymers exhibit more subdued fluctuations relative to their composite counterparts. The inherent uniformity of the polymer material results in more consistent cutting forces compared to the more variable forces observed in composites.

Discussion on the limitations of the cutting model: Firstly, although the Mulliken–Boyce model is effective for simulating low-speed cutting conditions, it may fail to fully capture the complex behavior at higher cutting speeds. At elevated velocities, the thermal-mechanical coupling effects at the tool-workpiece interface, including heat generation, lead to changes in material properties such as thermal softening and reduced cutting resistance, which are not fully incorporated in the current model. These effects may cause an underestimation of the cutting forces in the simulations. For example, at cutting speeds above 1 mm/s, the temperature rise in the cutting zone could reduce the yield strength of PMMA and CNTs, which is not considered in the current version of the Mulliken–Boyce model. Secondly, the absence of tool-chip adhesion effects in the model could explain some of the observed discrepancies. The interaction between the cutting tool and the material generates a shear force that leads to adhesion, especially under high-speed cutting conditions, where localized heating can cause significant adhesion between the tool and the chip. This phenomenon is not included in the current model, and its omission likely contributes to the underprediction of cutting forces. Thirdly, the model also disregards strain gradient effects, which are particularly important in nanosectioning operations. At small cutting depths, such as those used in this study (60–200 nm), the material undergoes significant plastic deformation over a small region, creating strain gradients. These gradients result in stress concentrations that significantly affect the cutting force but are not captured by the current model. Incorporating a more detailed strain gradient theory, such as a higher-order material model, could improve simulation accuracy.

**Figure 9:**
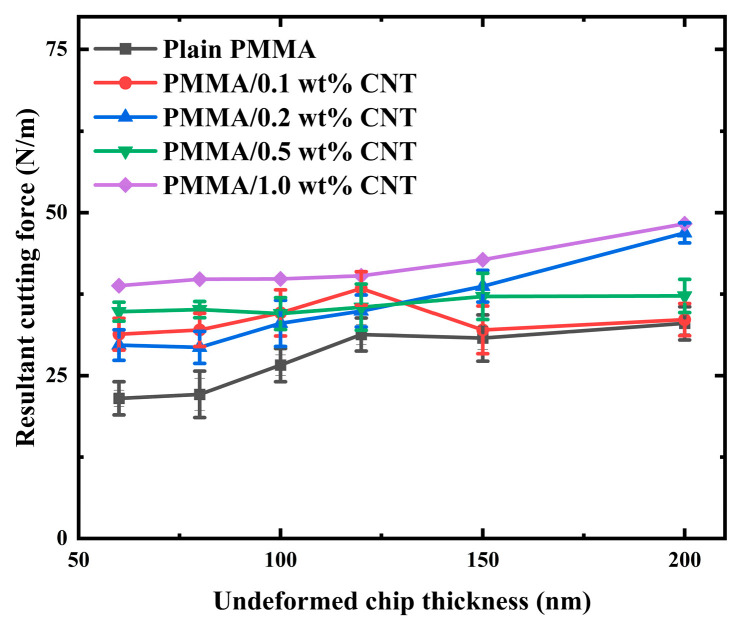
Simulated resultant cutting force of various PMMA/CNT nanocomposites and plain PMMA at various cutting thicknesses.

Figure 9 presents the simulated resultant cutting force for various PMMA/CNT nanocomposites in comparison to plain PMMA across different cutting thicknesses. The simulated cutting force for the PMMA/CNT nanocomposites is approximately 10% greater than that of the plain PMMA. However, there is a notable discrepancy between the simulated and experimental results: the simulated cutting force for the PMMA/CNT nanocomposites is roughly 50% less than the experimental results under identical cutting conditions. Specifically, for a cutting thickness of 200 nm, the simulated resultant cutting force for plain PMMA stands at about 30.4 N/m, while the experimental value is approximately 55.3 N/m. In the case of the PMMA/1.0 wt% CNT nanocomposite, the simulated value is about 35.7 N/m, contrasting with the experimental value of around 72.9 N/m. Three potential reasons can account for these discrepancies. First, the Mulliken–Boyce model might exhibit limitations when subjected to high-velocity loads. Second, the current model does not incorporate the adhesion effects observed at the chip-to-tool interface. Lastly, the model does not consider the influence of strain gradient effects [58].

### 5.2. Stress Distribution

**Figure 10:**
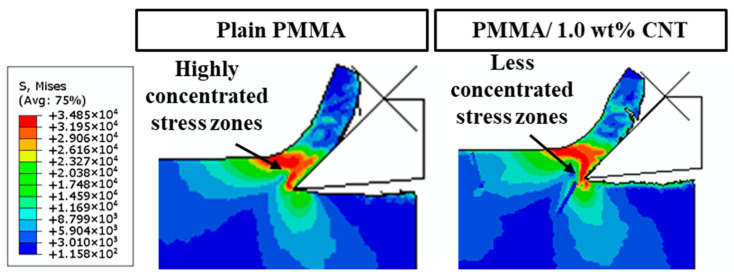
Stress distribution of plain PMMA and PMMA/1.0 wt% CNTs at 100 nm cutting thickness.

Figure 10 illustrates the stress distribution in plain PMMA compared to PMMA/1.0 wt% CNT at a cutting thickness of 100 nm. The plain PMMA exhibits pronounced stress concentrations within the tool-to-workpiece contact area. In contrast, the contact zone between the tool and the PMMA/CNT workpiece displays reduced stress concentration. During the initial cutting phase, CNTs experience elevated stress levels. However, as the tool progresses and the deformation of the base material intensifies, an increasing number of CNTs begin to encounter heightened stresses. This observation indicates that, during the cutting process, the CNTs bear significant stresses transferred from the base material.

**Figure 11:**
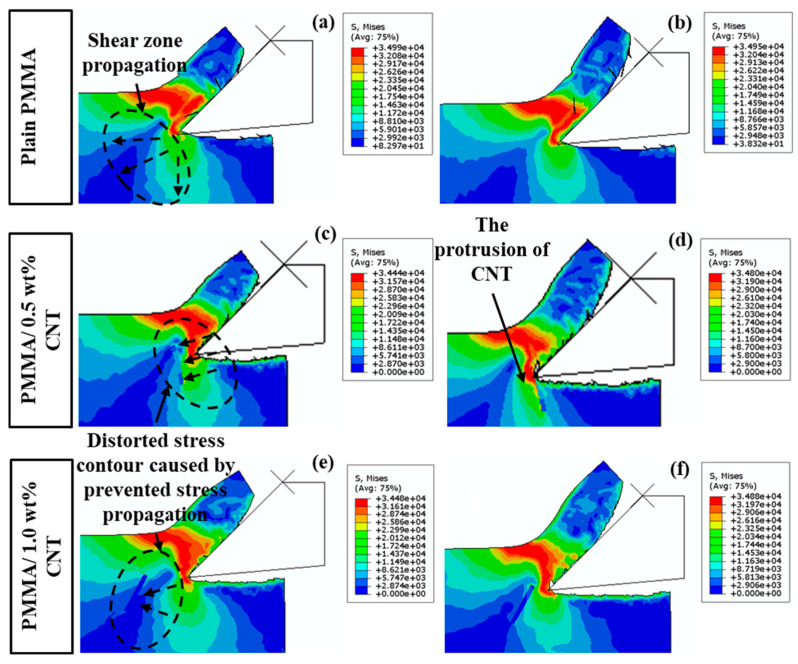
Stress distribution (S, Mises) at 60 nm cutting thickness: (**a**) plain PMMA—shear-zone propagation; (**b**) plain PMMA—edge stress localization; (**c**) PMMA/0.5 wt% CNTs—primary shear band growth; (**d**) PMMA/0.5 wt% CNTs—CNT protrusion at the interface; (**e**) PMMA/1.0 wt% CNTs—inhibited stress propagation; (**f**) PMMA/1.0 wt% CNTs—stress redistribution during chip flow.

Figure 11 illustrates the stress distribution in plain PMMA, PMMA/0.5 wt% CNTs, and PMMA/1.0 wt% CNTs at a cutting thickness of 60 nm. The incorporation of CNT introduces distinct phenomena, including the protrusion of CNT on the machined surfaces and sub-surface damage within the composites. This leads to a markedly different failure mechanism. Consequently, the machined surfaces of PMMA/CNT nanocomposites differ from those of plain PMMA. Furthermore, in composites with a higher concentration of CNTs, these CNTs not only offer enhanced reinforcement and mitigate stress propagation but also play a more pronounced role in chip formation, significantly affecting the morphology of the machined surface.

### 5.3. Validity and Limitations of the Geometric Approximation

To balance mechanistic insight with computational cost, the planar projection of three-dimensional MWCNTs is approximated here by two-dimensional rectangles. Random sequential adsorption (RSA) is used to generate configurations whose areal fraction and orientation statistics are consistent with a prescribed target volume fraction [59,60]. Although this abstraction does not explicitly represent cylindrical curvature, end effects, or size distributions, it can, at cutting thicknesses of 60–200 nm, capture the local stress transfer that occurs when a CNT encounters the tool and the corresponding relative variations in peak cutting force [61,62].

Numerical results show that, with geometric dimensions held fixed, merely increasing CNT content while maintaining random orientation reproduces the experimentally observed overall trend of higher cutting resistance and more pronounced fluctuations in the steady-state force. The lower force amplitude relative to experiments is consistent with this geometric approximation providing a conservative (lower-bound) estimate of local triaxiality and effective load-transfer length [63,64]. Accordingly, we regard the approximation as a feasible representation for trend assessment and mechanistic elucidation.

For higher quantitative fidelity, future work within the present framework will conduct parametric sensitivity studies—e.g., on aspect ratio, degree of orientational order, and interfacial cohesive parameters—together with uncertainty quantification [65]. These efforts will be integrated with existing SEM-based orientation statistics to delimit confidence bounds for the model predictions.

### 5.4. Experimental Validation

**Figure 12:**
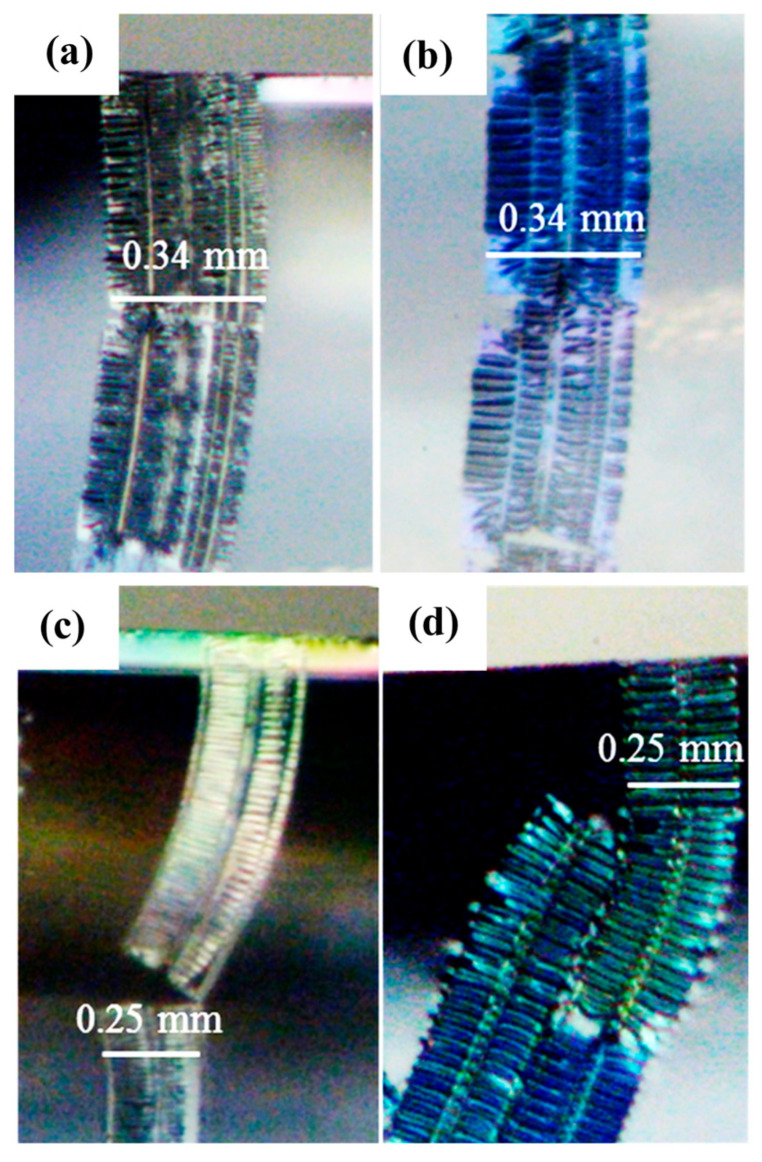
Images of chips of PMMA/CNT nanocomposites [69]: (**a**,**b**) PMMA/0.2 wt% CNTs sectioned at the cutting thickness of 60 nm and 200 nm, respectively, (**c**,**d**) PMMA/1.0 wt% CNTs sectioned at the cutting thickness of 60 nm and 200 nm, respectively.

Figure 12 presents images of chips derived from various PMMA/CNT nanocomposites. Further details of these chips have been elaborated upon in prior research [66]. Specifically, Figure 12a,b depict chips from the PMMA/0.2 wt% CNTs with cutting thicknesses of 60 nm and 200 nm, respectively. Conversely, Figure 12c,d represent chips from the PMMA/1.0 wt% CNTs, at cutting thicknesses of 60 nm and 200 nm. Theoretical considerations suggest a ductile-to-brittle transition in different composites. This transition implies the existence of a threshold: beyond this point, an increase in the CNT percentage renders the composite increasingly brittle, increasing its propensity for failure during the cutting process [67]. Notably, both the plain PMMA and the PMMA/CNT nanocomposites consistently produced continuous chips across experiments and simulations. This observation aligns with findings by Satapathy et al. [68], who identified a ductile-to-brittle transition at a CNT loading of 4.0 wt%. The cutting process, in its intricacy, offers valuable insights into the onset of damage phenomena, which can, in turn, influence the chip formation process. Consequently, it can be inferred that the 1.0 wt% CNT addition does not surpass the critical threshold that would alter chip formation.

## 6. Conclusions

This study provides an analysis of the nanosectioning behavior of PMMA/CNT nanocomposites using a microstructural-level FE model. The analysis reveals that introducing 1.0 wt% CNTs does not surpass the critical threshold necessary to alter chip formation mechanisms, indicating that the nanocomposite maintains similar chip formation characteristics to plain PMMA at this CNT concentration. Any interaction between a specific CNT and the cutting tool is likely to result in an increased cutting force. Specifically, when 1.0 wt% CNTs are added to PMMA, the cutting force increases from approximately 55.3 N/m to 72.9 N/m, which corresponds to approximately a 31.8% increase. While the primary trends in strain distribution in PMMA/CNT composites closely match those observed in plain PMMA, discernible differences between the two materials persist, highlighting the impact of CNT inclusion on the sectioning response. Furthermore, the incorporation of CNTs affects specific phenomena, notably causing protrusion of CNTs on machined surfaces and leading to sub-surface damage within the composites.

## Figures and Tables

**Table 1:** Epoxy material parameters defined in the Mulliken–Boyce model [41].

Symbol	Unit	**Value**
${\dot{\gamma }}_{0,a}$	$10^{15}\;s^{-1}$	6.95
${\dot{\gamma }}_{0,\beta }$	$10^{3}\;s^{-1}$	1.77
$\Delta G_{\alpha }$	$10^{-18}\;J$	5.528
$\Delta G_{\beta }$	$10^{-20}\;J$	6.036
$\alpha_{p,\alpha }$		0.26
$\alpha_{p,\beta }$		0.26
$h_{\alpha }$	MPa	200
$h_{\beta }$	MPa	500
$C_{R}$	MPa	14
N	$m^{-\frac{1}{2}}$	2.1

**Table 2:** Cutting model and condition parameters used in the FE cutting model.

Parameter	Value
Cutting speed	1.0 mm/s
Cutting thickness	60, 80, 100, 120, 150, 200 nm
Tool rake angle	45.0°
Tool clearance angle	5.0°
Content of nanoparticles	0.1 wt%, 0.2 wt%, 0.5 wt%, 1.0 wt%
Surface area fraction of nanoparticles	0.05%, 0.1%, 0.25%, 0.5%
Length of nanoparticle	100 nm
Width of nanoparticle	10 nm
Length of model	1.6 µm
Height of model	0.5 µm

## Data Availability

The original contributions presented in this study are included in the article. Further inquiries can be directed to the corresponding author.
